# Quantum Cone—A Nano-Source of Light with Dispersive Spectrum Distributed along Height and in Time

**DOI:** 10.3390/nano14191580

**Published:** 2024-09-30

**Authors:** Arturs Medvids, Patrik Ščajev, Kazuhiko Hara

**Affiliations:** 1 Insitute of Technical Physics, Riga Technical University, Paula Valdena 3/7, 1048 Riga, Latvia; 2 Institute of Photonics and Nanotechnology, Faculty of Physics, Vilnius University, Saulėtekio Ave. 3, 10257 Vilnius, Lithuania; patrik.scajev@ff.vu.lt; 3 Research Institute of Electronics, Shizuoka University, 3-5-1 Johoku, Naka-ku, Hamamatsu 432-8561, Japan; hara.kazuhiko@shizuoka.ac.jp

**Keywords:** quantum cone, stretched exponential law, photoluminescence lifetime, nano-source of light

## Abstract

We study a quantum cone, a novel structure composed of multiple quantum dots with gradually decreasing diameters from the base to the top. The dot distribution leads to a dispersive radiated spectrum. The blue edge of the spectrum is determined by the quantum confinement of excitons on top of the cones, while the red edge is determined by the bandgap of a semiconductor. We observe the kinetics of photoluminescence by obeying the stretch-exponential law from quantum cones formed on the surface of diamond-like carbon (DLC). They are explained by an increase in the lifetime of excitons along the height of the cone from the top to the base of the cone and an increasing concentration of excitons at the base due to their drift in the quasi-built-in electric field of the quantum cone. The possible visualization of the quantum cone tops of DLC using irradiation by a UV light source is shown. A quantum cone is an innovative nano-source of light because it substitutes for two elements in a conventional spectrometer: a source of light and a dispersive element—an ultrafast monochromator. These features enable the building of a nano-spectrometer to measure the absorbance spectra of virus and molecule particles.

## 1. Introduction

Diamond-like carbon (DLC) films have recently attracted considerable attention as a promising material for constructing luminescent emission devices [1,2,3,4]. The prospect of these devices is based on the following properties of DLC films: the capability of synthesizing DLC structures with an altering optical bandgap (up to 4 eV) [5], intensive visible and UV luminescence, the possibility of the doping of DLC with different kind of impurities [6], high chemical and radiation hardness [7], and the novel mechanically durable DLC coatings [8]. On the other hand, the tendency to reduce the size of artificial light sources allowed for the use of such sources in microelectronics, medicine, and biology to study micro-objects. At the same time, the requirements for improving the quality of such sources as: increase in the intensity, the expansion of the spectrum of radiation, its uniformity, and the controllability of its parameters over time, have increased. The importance of this trend is confirmed by the appearance of international conferences and monographs in this direction. Spectroscopy research of microscopic objects, for example, microbes, molecules, and blood cells, requires non-traditional research methods, for example, the use of an optical probe [9] because the size of such objects is smaller than the wavelength of visible light; therefore, the micro-object neither absorbs nor scatters it effectively. The study of a micro-object requires a light source much smaller than the object itself, such as quantum dots—0D. Therefore, the award of the 2023 Nobel Prize in Chemistry to Munga J. Bawendi, Louis E. Brus, and Alexei I. Ekimov “for the discovery and synthesis of quantum dots” confirms that the quantum confinement effect is a major focus in solid state physics today [10,11,12]. Therefore, developing new optical and electronic devices and technologies based on quantum dots is one of the main tasks for scientists nowadays.

As known [13], a quantum dot has one line in the luminescence spectrum, the so-called monochromatic radiation. A new quantum system called quantum cones [14] on the surface of a Ge single crystal irradiated by an Nd:YAG laser was found. These cones possess unique optical properties: a huge “blue shift” of the photoluminescence (PL) spectrum by 1.1 eV with an increased intensity of more than a million times. However, the narrow radiation spectrum of a single quantum dot is a disadvantage for use in spectroscopy. On the basis of our previous investigations of Quantum Cones (QCs) on the irradiated surface of a Si single crystal [15] and a SiGe solid solution [16] formed by laser radiation, we propose a new nano-source of light with unbelievable optical and photoelectrical properties, such as dispersive spectrum radiation due to the graded bandgap of QC [17,18,19] and an ultrafast controlled change of the spectrum in time. Therefore, the research aims to develop a new nano-source of light with a dispersive spectrum distributed in time and space.

## 2. Materials and Methods

**Figure 1:**
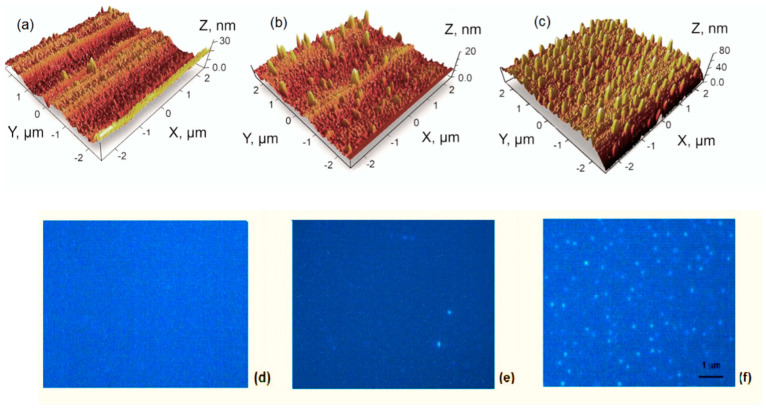
AFM images of DLC samples: 1A (**a**), 2A (**b**), and 3A (**c**) surfaces [3] and the fluorescent microscope images of 1A (**d**), 2A (**e**), and 3A (**f**) at UV excitation for the same surfaces.

The DLC films with nano-cones were formed by magnetron sputtering of a C precursor hexamethyldisiloxane (Fluka, 98%) on the Si (001) substrate and subsequent thermal annealing at *T* = 1050 °C temperatures for 60 min in a nitrogen atmosphere: sample 1A had thickness *d* = 240 nm, 2A − *d* = 600 nm, and 3A − *d* = 640 nm [3]. Raman spectra and XRD analysis confirmed the DLC composition of the films and the nano-cones [3]. The Raman D-band intensity was 2.0 times larger than that of the G-band, indicating a high amount of sp^3^ bonding. The AFM measurements, shown in Figure 1a–c, indicate that the thickness of the carbon film plays a vital role in the nano-cone formation. Therefore, the height of the cones and their concentration on the surface of sample 3A in Figure 1c is more than that on sample 2A in Figure 1b, while on the surface of sample 1A (Figure 1a), the cones are almost absent. The nano-cones in 3A have a base of 120 nm and a height of 60 nm, while in 2A, the base is 200 nm, and height varies in the 5–20 nm range.

**Figure 2:**
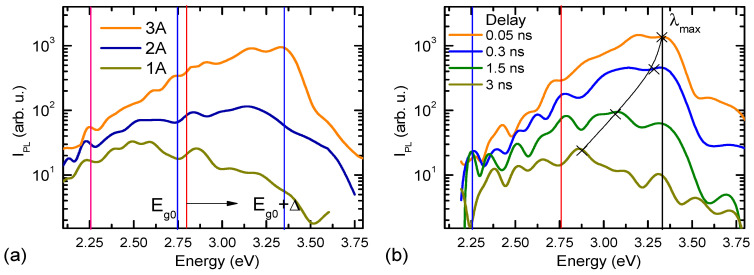
(**a**) PL spectra of DLC samples 1A, 2A, and 3A. (**b**) PL spectra of sample 3A with a time delay of 0.05 ns, 0.3 ns, 1.5 ns, and 3 ns. The spectral resolution is 24 meV. The excitation fluence is 1.2 mJ/cm^2^, and the wavelength is 213 nm.

Optical images of 1A, 2A, and 3A sample surfaces irradiated by a UV light source (laser with *λ* = 405 nm; excitation blocking filter BLP01; microscope objective: 100×, NA—0.9) are shown in Figure 1d–f. The image of the 1A sample surface is homogenous, but the bright points on the surfaces of samples 2A and 3A arise, especially on the surface of 3A. This visualization of the DLC quantum cones by means of irradiation with an ultraviolet light source makes it possible to estimate the diameter by the height of the cone and, in any case, the position of the upper quantum point. The visualization for DLC is problematic because the blue part of the PL spectrum of sample 3A is located at quantum energies of 3.31 eV and above, as shown in Figure 2. We can only see the blue color and conclude that the diameter is about 2 nm according to Figure 2, using *E_max_* and the formula from the paper [13]. Our experiments on diamond-like carbon (DLC) films with cones on the surface have shown that the optical properties of the cones are the same as those formed by laser radiation on surfaces of Si, Ge, and CdTe [14,15,16,17,18,19], for example, the “blue shift” of the PL spectra and the “red shift” of the Raman spectrum.

Therefore, studying the PL kinetics of DLC films with nano-cones is very important for understanding the features of the exciton behavior, such as the recombination rate and drift of the excitons in the quantum cones. Such data will make it possible to estimate the dynamic parameters of nano-light sources: the linear dispersion and the rate of the spectral shift.

## 3. Results

The time-resolved PL measurements were obtained using a Hamamatsu streak camera (C10627) attached to an Acton monochromator. Excitation was performed by 213 nm and 200 fs duration pulses obtained as a second harmonic of 423 nm pulses generated by PHAROS laser in the ORPHEUS (Light Conversion) parametric generator at a 10-kHz frequency. The scattered excitation light was blocked by a long-pass filter.

The sharpest cones in sample 3A provide the mostly blue-shifted PL spectrum (Figure 2a). Sample 1A has the dominant DLC layer emission with a peak at 500 nm (2.5 eV). Higher cone density provides stronger emission in the 2.75–3.5 eV range. Due to quantum confinement in the cone, the bandgap increased on its top and in its quantum energy levels; we ascribe these energies to quantum dots (nano-spheres) with different diameters to simplify the analysis of the experimental data. The calculation of the nano-sphere diameter *d* at the top of the cone provides 2.1 nm if using a spectral blue-shift (Δ) Formula (1) for a spherical nanoparticle according to [13]:

(1) $$E_{g}=E_{g0}+\Delta ,\;\Delta =1.42\frac{{\left(\pi \hbar \right)}^{2}}{\mu d^{2}}.$$

Here, *E_g_* is the blue-shifted bandgap due to the quantum confinement, *E_g_*_0_ is the base material (DLC) indirect bandgap (we assume 2.8 eV, similarly as determined in [20] where the D to G intensity ratio was 2), *μ* = 0.43 m_0_ is the reduced effective mass of the electron-hole pairs (EHPs) [21], assuming that the electron and hole masses are equal in DLC (these masses are equal in diamond semiconductor [22]), and Δ ~ 0.56 eV is the blue shift of the PL spectrum of the 3A sample obtained by comparing it with the PL spectrum of the 1A sample (Figure 2a).

**Figure 3:**
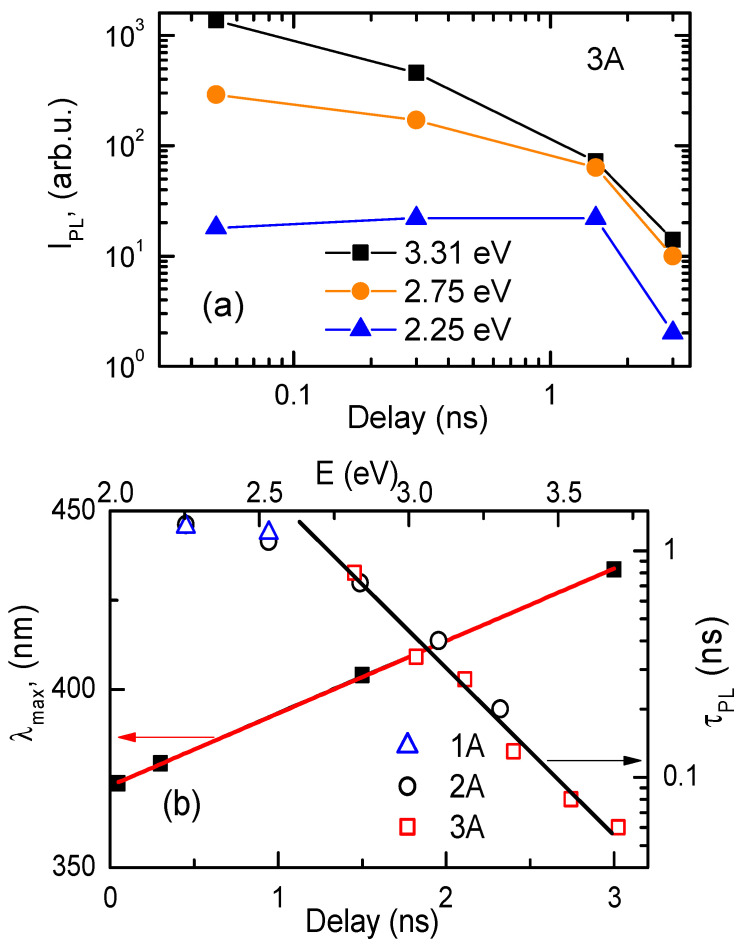
(**a**) PL intensity dependence on the delay time at *E* = 3.31 eV, 2.75 eV, and 2.30 eV emission energies. (**b**) The peak PL emission wavelength dependence on the delay time for the 3A sample and the PL decay time dependence on the emission quantum energy (top scale); the solid lines show that *λ_max_* (Delay) = 372 nm + 20 nm/ns × Delay and *τ_PL_*(*E*) = 4000 ns × exp(−*E*/*E*_0_) with *E*_0_ = 0.33 eV, respectively. The rate of spectra change is *V_λ_* = 3 × 10^10^ nm/s.

Figure 2b demonstrates that the intensity of the PL spectra of the DLC sample 3A depends on the time delay of 0.05 ns, 0.3 ns, 1.5 ns, and 3 ns, which is an unusual behavior. The maximum intensities of the PL spectra are shifted to the red part of the spectra with time, as shown by the thin black line in Figure 2b. However, the maximum PL spectrum for sample 1A is usually at *E* = 2.5 eV and does not change position with time. The former peculiarity is explained by the drift of EHPs in a quasi-built-in electric field from the top to the base of the cone in a graded bandgap structure [23]. Evidence of this phenomenon is demonstrated in Figure 3a’s blue curve. The recombination velocity of EHPs with a delay time is significant for a photon energy of 3.31 eV, slow for 2.75 eV, and it becomes negative for 2.25 eV (at least in the region from 0.05 ns to 1.5 ns). This means that an additional concentration of EHPs appears at the base of the cone after laser irradiation of the sample due to their drift downward. The peak PL emission wavelength *λ_max_* consequently shifts linearly with the delay time, as shown in Figure 3b.

**Figure 4:**
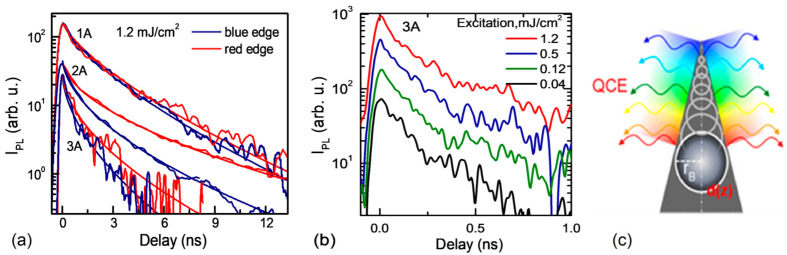
(**a**) PL kinetics of samples 3A, 2A, and 1A on the edges of PL spectra at 1.2 mJ/cm^2^ excitation and 0.3 eV spectral integration range; the blue edge and red edge correspond to 3.31 eV and 2.75 eV central energies (for 2A and 3A), 2.75 eV and 2.25 eV for 1A, respectively; the curves are vertically shifted for clarity. (**b**) Spectrally integrated PL kinetics of sample 3A at different excitations. (**c**) The scheme of the quantum cone emission.

A comparison of the PL decay profiles and rates for the three samples at the highest energy of a photon quantum (“blue edge”) and the lowest energy (“red edge”) at the top and the base of the cone, respectively, is shown in Figure 4a. These curves coincide only for sample 1A; still, for samples 2A and 3A, the “red edges” provide higher *I_PL_* values in comparison with the “blue edges” at longer delays. The fitting formula for PL kinetics *I*_*PL*_ = *I*_0_ × exp (−(*t*/*τ*_0_)^*β*^) is the stretch exponent, and the corresponding parameters are listed in Table 1. Sample 3A features the sharpest cones and demonstrates the shortest decay time *τ*_0_ = 0.35 ns and similar *β* = 0.55 in comparison to sample 2A (*τ*_0_ = 0.59 ns and *β* = 0.56). Such differences in the PL kinetics are explained by the difference in the sharpness of the cones (the sharpness of the cone is the ratio of its height to the base), which is responsible for the diameter of the quantum dot at the top of the cone [17], and therefore, for *τ*_0_, it depends on the confinement volume [24]. Each nano-cone is composed of multiple quantum dots, with decay time becoming shorter and a smaller radius approaching the top of the cone. Therefore, a decrease in the nano-cone top radius induces the appearance of more blue-shifted components with faster decay. The decay time for a fixed quantum dot in the nano-cone follows the same exponential tendency for both 2A and 3A in the 2.8–3.7 eV range (from Figure 3b, we find *τ_PL_*(*E*) = 4000 ns × exp(−*E*/*E*_0_), with *E*_0_ = 0.33 eV—the potential on the cone top, which can correspond to the built-in quasi-electric field (*dE_g_*/*dz*) causing the EHPs drift towards the cone base). A deviation at the lowest energies is observed when the DLC layer indirect band-edge emission starts to dominate the PL kinetics with a ~1.3 ns decay time (see Figure 3b). Therefore, the stretch exponent law applies to the PL kinetics of any individual nano-cone due to *τ*_0_ increasing gradually from the top to the base of the cone. The possible variations in the nano-cone tip dimensions indicate that the fitted stretch component is averaged through the nano-cone ensemble.

The decays of the nano-cones in 3A were found to be excitation-independent (Figure 4b), together with excitation-independent PL spectra, which confirms that the decay shape is not affected by any nonlinear process (e.g., Auger recombination). The near-coincidence of both spectral edge curves for sample 1A means that the PL kinetics obey the exponential law without the presence of the cones. In the latter case, the slight nonlinearity of the decay can be explained by the inhomogeneous distribution of defects in the DLC film of 1A (i.e., structural disorder [3,24]). In the literature on the kinetics of PL, the stretch exponent law is often used to explain the disordered system’s properties [25]. On the other hand, the crystallinity of samples 2A and 3A are very ordered, as confirmed by the presence of the second harmonic of G and D lines in Raman spectra [3]. Corresponding to theory [26], the smallest excitons are at the top of every cone [27], and the size of the excitons gradually increases to the base of the cone (Figure 4c).

The parameters of the quantum cones as sources of light are in the spectral range of Δ*λ* = 350–550 nm; the active part of the nano-cone *h* = 16 nm; the linear dispersion of the quantum cone *dh*/*d*λ = 0.1 (a height 0.1 nm contains the spectral range of 1 nm); and the shortest relaxation time *τ* = 20 ps. The PL decay time’s dependence on the emission energy is *τ_PL_*(*E*) = 4000 ns × exp(−*E*/*E*_0_), *E*_0_ = 0.33 eV. The rate of the spectra change is *V_λ_* = 3 × 10^10^ nm/s. It is an ultra-fast nano-monochromator.

## 4. Discussion

The expression nano-source of light or nano-light source is often used in the literature [28] due to the study of nanostructures, and elaborating nanotechnology is the mainstream in up-to-date science. Very often, the nano changes nothing in fundamental physics compared with the micro, and only decreases the light source’s volume [29]. Still, our quantum cone is a fundamentally new type of nano-source of light because the quantum cone is a light source with a dispersion spectrum distributed along the coordinate and time due to the quantum confinement effect [10,11,12,13]. Possible applications of the quantum cone as a nano-source of light are as follows: it can be the main part of a spectrometer–monochromator. Such a light source would replace two elements in a conventional spectrometer—a light source and a dispersive element. Another application of this research is in the visualization and estimation of the diameter of the invisible tip of the nano-cone, as shown in Figure 1c,f. For example, this is an essential task for the user and manufacturer when measuring a cantilever’s diameter in an atomic force microscope.

## 5. Conclusions

In summary, we showed the possibility of constructing a nano-source of light—a quantum cone with a dispersive spectrum distributed in time and along the cone height. The spectral range of the dispersive spectrum depends on the quantum cone’s parameters and composition. The blue edge of the spectrum is determined by the quantum confinement of excitons on top of the cones, while the red edge is determined by the bandgap of a semiconductor.

The kinetics of photoluminescence from cones obey the stretch exponential law and is explained by the increase in the lifetime of excitons with the height of the cone from the top to the base of the cone as well as the increasing concentration of excitons at the base due to their drift in the quasi-built-in electric field of the quantum cone.

Visualizing and estimating the diameter of a non-visible nano-cone’s top is an essential task for the user and producer, for example, the diameter of a cantilever in an atomic-force microscope. A quantum cone as a nano-source of light allows for the building of a nano-spectrometer to measure the absorption spectrum of individual nanoparticles or viruses.

A quantum cone is a new type of nano-source of light because it substitutes for two elements in a conventional monochromator: the light source and the dispersive element. Moreover, it is an ultra-fast nano-monochromator.

## Figures and Tables

**Table 1:** Exponential fitting parameters for the studied samples.

	Cone Density	Red Edge		Blue Edge	
Sample	μm^−2^	*τ*_0_, ns	*β*	*τ*_0_, ns	*β*
3A	11	0.52	0.55	0.35	0.55
2A	3.1	1.12	0.56	0.59	0.56
1A	0.2	1.35	0.72	1.25	0.72

## Data Availability

The data that support the findings of this study are available upon reasonable request from the authors.
